# *TP53* Mutational Status Is a Potential Marker for Risk Stratification in Wilms Tumour with Diffuse Anaplasia

**DOI:** 10.1371/journal.pone.0109924

**Published:** 2014-10-14

**Authors:** Mariana Maschietto, Richard D. Williams, Tasnim Chagtai, Sergey D. Popov, Neil J. Sebire, Gordan Vujanic, Elizabeth Perlman, James R. Anderson, Paul Grundy, Jeffrey S. Dome, Kathy Pritchard-Jones

**Affiliations:** 1 Cancer Section, Institute of Child Health, University College London, London, United Kingdom; 2 Divisions of Molecular Pathology and Cancer Therapeutics, Institute of Cancer Research, London, United Kingdom; 3 Departments of Histopathology, Great Ormond Street Hospital, London, United Kingdom; 4 Department of Pathology, School of Medicine, Cardiff University, Cardiff, United Kingdom; 5 Department of Pathology, Ann and Robert H. Lurie Children's Hospital, Chicago, Illinois, United States of America; 6 Department of Biostatistics, College of Public Health, University of Nebraska Medical Center, Omaha, Nebraska, United States of America; 7 Departments of Pediatrics and of Oncology, University of Alberta, Edmonton, Alberta, Canada; 8 Division of Pediatric Hematology/Oncology, Children's National Medical Center, Washington, District of Columbia, United States of America; University of Bristol, United Kingdom

## Abstract

**Purpose:**

The presence of diffuse anaplasia in Wilms tumours (DAWT) is associated with *TP53* mutations and poor outcome. As patients receive intensified treatment, we sought to identify whether *TP53* mutational status confers additional prognostic information.

**Patients and Methods:**

We studied 40 patients with DAWT with anaplasia in the tissue from which DNA was extracted and analysed for *TP53* mutations and 17p loss. The majority of cases were profiled by copy number (n = 32) and gene expression (n = 36) arrays. *TP53* mutational status was correlated with patient event-free and overall survival, genomic copy number instability and gene expression profiling.

**Results:**

From the 40 cases, 22 (55%) had *TP53* mutations (2 detected only after deep-sequencing), 20 of which also had 17p loss (91%); 18 (45%) cases had no detectable mutation but three had 17p loss. Tumours with *TP53* mutations and/or 17p loss (n = 25) had an increased risk of recurrence as a first event (p = 0.03, hazard ratio (HR), 3.89; 95% confidence interval (CI), 1.26–16.0) and death (p = 0.04, HR, 4.95; 95% CI, 1.36–31.7) compared to tumours lacking *TP53* abnormalities. DAWT carrying *TP53* mutations showed increased copy number alterations compared to those with wild-type, suggesting a more unstable genome (p = 0.03). These tumours showed deregulation of genes associated with cell cycle and DNA repair biological processes.

**Conclusion:**

This study provides evidence that *TP53* mutational analysis improves risk stratification in DAWT. This requires validation in an independent cohort before clinical use as a biomarker.

## Introduction

Wilms tumour (WT) or nephroblastoma, the most frequent renal tumour of childhood, affects around 1 in 10,000 children before the age of 15 years. Most patients with WT in the Western world are treated within prospective clinical trials conducted by either the International Society of Paediatric Oncology - Renal Tumor Study Group (SIOP-RTSG, Europe) or the Children's Oncology Group (COG, North America) that each report long-term survival rates of over 85% [1]–[8]. However, a substantial minority (∼25%) respond poorly or relapse with current therapies and approximately 50% of these children will succumb to their tumour despite intensive re-treatment [9], [10].

Risk stratification is largely based on tumour stage and histology which is key to improving clinical management; 4–10% of WT display anaplasia, which is defined morphologically by the presence of cells with at least threefold nuclear enlargement, hyperchromasia and abnormal mitotic figures [11]. A further distinction is made between focal (FA) and diffuse (DA) anaplasia, based on the topographic distribution of anaplastic elements within the tumour [12]. Presence of DA is the most important adverse prognostic indicator in pre-treated (SIOP) and in chemotherapy-naïve tumours (COG) and patients are assigned to more intensive treatment [13], [14].

*TP53* mutations are only found in the anaplastic areas of WT [15], suggesting an association between *TP53* and anaplastic cells. Only small numbers of cases of DAWT have been analysed for *TP53* mutations, usually as a subgroup of a larger dataset of WT, reflecting the rarity of this histological type. Four studies have clearly analysed 14 DAWT, 12 (86%) displayed mutations in *TP53* [15]–[18]. *TP53* mutations were also evaluated in anaplastic WT (AWT) without further classification [19]. Although *TP53* mutation seems to be predictive of response to treatment and patient survival in several cancers, studies that used immunohistochemistry to investigate p53 prognostic value yielded inconsistent results [20], leading to the conclusion that this technique alone is not suitable for assessing *TP53* mutational status. Immunostaining for p53 is positive in ∼76% of anaplastic WT and ∼8% of favourable histology WT - those with ‘nuclear unrest’ which harbour only some of the morphological criteria to be considered anaplastic [21], [22]. In pre-treated patients, p53 positivity was associated to high risk cases that relapsed [23].

Previously, we showed 17p loss in a significant proportion of AWT [24]. Since only small cohorts of DAWT were previously evaluated for *TP53* mutation and its clinical implications could therefore not be rigorously assessed, we brought together the largest cohort of anaplastic WT analysed to date to investigate whether the generally accepted frequency of *TP53* mutations is correct, and to determine the relationship between *TP53* status and patient outcome.

## Methods

### Patients and samples

Forty patients with unilateral DAWT were included in this analysis. The inclusion criteria were: Wilms tumour with diffuse anaplasia confirmed by central pathology review according to standard international criteria [12] *and* presence of anaplastic features in the frozen tumour specimen used to extract DNA. Thirty-two cases came from the COG tumour bank and eight from the UK Children's Cancer and Leukaemia tumour bank. All 32 patients from COG were enrolled on the National Wilms Tumor Study-5 protocol (08/1995 to 05/2002) [13] and eight patients were treated in the SIOP WT 2001 trial (02/2002 to 12/2011). All patients were treated according to the high risk arm of their respective trials (**Tables S1 and S2**).

The frozen tumour samples were taken from a larger cohort of 51 DAWT similarly subject to central pathology review. However 11 tumours were excluded from analyses either because they lacked evidence of anaplasia in the frozen specimen (n = 6) or it was not possible to clearly categorise the haematoxylin-eosin section of the frozen specimen (n = 5).

All samples were obtained from patients whose parent or legal guardian had provided written informed consent for research use (or for older patients, the patient themselves had provided written consent or assent), in accordance with national regulations. Ethical approval for the study was given by East Midlands - Derby Research Ethics Committee (National Research Ethics Service (NRES) in the United Kingdom) with reference approval number MREC/01/4/086 given on 17^th^ January 2002.

### Sequencing and analysis of *TP53*

Genomic DNA was extracted using proteinase K digestion followed by phenol/chloroform extraction as described by manufacture's recommendations. Primer sequences, product lengths and PCR conditions for the 11 exons of *TP53* were retrieved from the IARC TP53 website [20]. Sanger sequencing was performed using the BigDye Terminator v3.1 Cycle Sequencing Kit (Applied Biosystems) according to the manufacturer's protocol using an ABI PRISM 3130 Genetic Analyzer (Applied Biosystems). Sequences were analysed using Mutation Surveyor software v3.20 (SoftGenetics).

Samples negative for *TP53* mutations by Sanger sequencing were deep-sequenced to detect mutations present in a minority cell population. Indexed libraries of the PCR products were prepared using the Illumina Nextera XT kit with supplied protocol and sequenced using an Illumina MiSeq instrument, generating 2×150 bp reads with a mean coverage of 162x. Only sequences that passed quality control were analysed further. All sequence alterations were reviewed in the Integrative Genomics Viewer (IGV) version 2.3 [25] and classified as missense, nonsense frameshift, in frame duplication, in frame deletion or splicing mutations by ANNOVAR [26].

### Multiplex Ligation-dependent Probe Amplification (MLPA) analysis

Chromosome 17p copy number, where *TP53* is located, was assessed by a MLPA assay (P380-X2 custom probemix, MRC-Holland b.v.) according to the manufacturer's protocol (http://www.mrc-holland.com) using 100 ng of DNA (Qubit) with A260/A230 ≥1 (Nanodrop) and containing ≥10 mM Tris-HCl pH 8.2 was used. Data normalisation and analysis was performed with Genemarker v1.85 (Softgenetics).

### Analyses of Copy Number and Gene Expression using Microarrays

To infer genomic instability and to confirm 17p loss identified by MLPA, we used genomic copy number BAC arrays from our previously reported data set [24], which has an overlap of 32 patients with the 40 patients from this study. In summary, cases were co-hybridized against unmatched, unrelated pooled normal DNA on the Breakthrough Breast Cancer Research Centre 32k BAC tiling path CGH array. Copy number alterations were calculated based on circular binary segmentation [27], [28] followed by merging of adjacent segments that did not differ significantly in copy number [29], as implemented in the Bioconductor packages DNAcopy and aCGH (http://www.bioconductor.org). Genomic instability was defined in the case of an alternating status in the level of copy number alterations (CNA) [30], [31] using three parameters: 1. More than two contiguous BAC probes with the same copy number, 2. Size range between 1 Kb and 100 Mb to exclude non-focal CNA and 3. Segmentation predicted ≤0.1 for loss and ≥0.1 for gain.

From the same study [24], we retrieved gene expression profiles of 36 cases that also overlap with the 40 patients from this study, and identified differences in gene expression between samples with wild-type and mutated *TP53*. In summary, tumour and reference cDNA (Universal Human Reference cDNA (Stratagene, La Jolla, CA) were co-hybridized to the Breakthrough Breast Cancer Research Centre 17k 2.1.2 cDNA microarray (ArrayExpress accession number A-MEXP-259). Data are available at GEO (GSE609400).

### Statistical analyses

Event-free survival (EFS) was defined to be the time from diagnosis to disease recurrence or other disease related event (including death as a first event from any cause), both clinically identified. Overall survival (OS) analysis included cancer-related deaths, one case where death resulted from infection and one case from toxicity (both had suffered relapse as a first event); the survival interval was calculated relative to the date of diagnosis. Estimates of time-to-event distributions were calculated using the Kaplan-Meier method and differences compared using the log-rank test. Estimates of the relative risk of events between patient subsets were estimated using the Cox regression model. Patients were right censored due to end of study or loss to follow up.

These analyses were performed using IBM SPSS version 21 for Windows and SAS version 9.3. Two-tailed student's t-test assuming unequal variance (given by Levene's test) was used to compare the quantity of copy number alterations between two groups: with wild-type or mutant *TP53*.

All results were considered significant when p-values were less than 0.05.

Expression analyses were performed using BRB-ArrayTools developed by Dr. Richard Simon and the BRB-ArrayTools Development Team. The BRB-ArrayTools was used to identify differentially expressed genes between wild-type and mutated *TP53*, and for gene ontology analyses. After a LOWESS normalization procedure, the class comparison tool uses a Two-sample T-test to find discriminating genes and to confirm their statistical significance p≤0.01. We compared tumours with mut*TP53* versus wt*TP53*. Biological processes were considered over-represented when 1) at least five genes were classified in that process and 2) an observed vs. expected' ratio of >2-fold was observed.

## Results

### Characterization of *TP53* in the tumours

**Figure 1:**
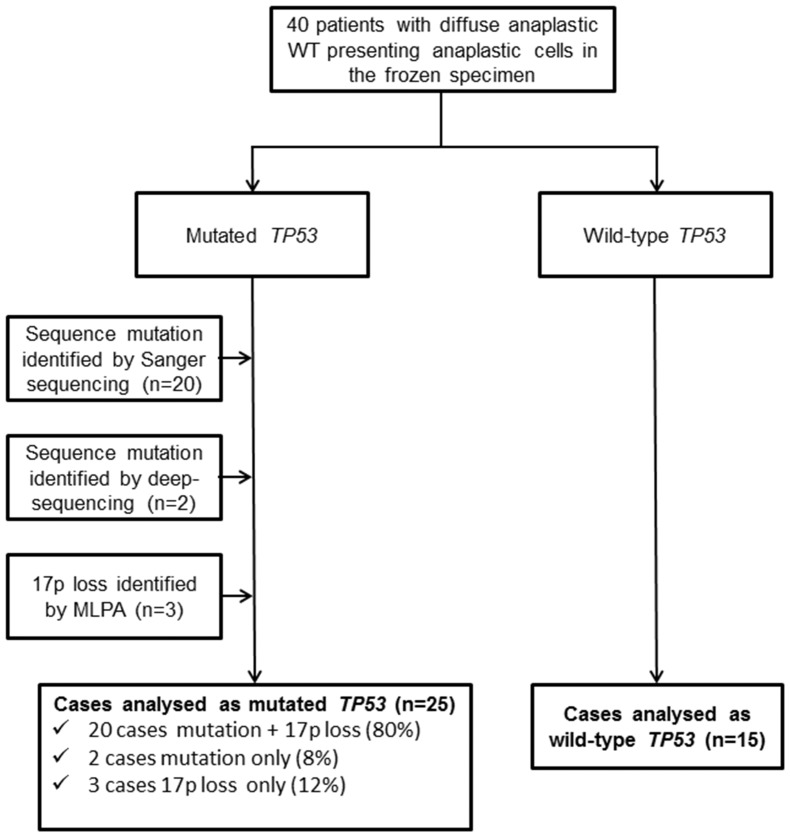
Consort diagram showing the 40 cases of diffuse anaplastic Wilms tumour used for the survival analysis by *TP53* mutation and/or 17p loss.

All samples were screened for *TP53* mutations in exons 1 to 11, including intron-exon boundaries, with reference to the published wild type sequence (Chr 17 NC_000017.9, **Table S3**). Distribution of cases with respect to *TP53* status is displayed in **Figure 1**.

Due to the rarity of DAWT, samples were pooled from two major tumour banks (NWTSG-5 and UK SIOP 2001). There were 51 patients with DA confirmed by central review from which we used 40 for further analyses. For the 40 patients, the median age at diagnosis was 4.6 years (1.42 to 9.17 years, which is older than reported for non-anaplastic WT [32]. Only one patient was aged less than 2 years at diagnosis and did not relapse. The stage distribution was 11 stage I, (27.5%); 8 stage II (20%), 16 stage III (40%); 5 stage IV (12.5%).

Of the 40 cases, 20 (50%) had at least one *TP53* mutation detected by Sanger sequencing; one case had two mutations (**Table 1**). In two further cases, *TP53* mutations were only detected by deep sequencing (162-fold). The pathology review noted that DA within the tumour was in the middle of a larger proportion of fat and necrotic cells in one case but the other had a classical presentation of DA. One case (1136) had mutation already described in AWT [16] (Table 1).

**Table 1:** Cases of diffuse anaplastic WT with *TP53* mutations.

Sample	Trial	Copy number at *TP53* locus	Exon	Mutation (annotation in Hg19)
*Frameshift deletion*				
1149	COG	Loss	10	g.7573999-7574008het_delTGTTCCGAGA
3036	COG	Loss	10	g.7574000-7574006delTTCCGAG
3048	COG	Loss	10	g.7574013-7574013het_delC
*Frameshift insertion*				
3477	SIOP	Loss	11	g.7572933-7572933dupA
*Nonframeshift deletion*				
2557	SIOP	Loss	5	g.7578447-7578447dupCATCTACAAGCAGTCACAGCACATGAC
1144	COG	Loss	6	g.7578275-7578277delCTC
1133	COG	Loss	10	g.7574026-7574026dupGGCGTG
*Nonsynonymous SNV*				
3044	COG	Loss	5	g.7578406-7578406G>GA¥
3047	COG	Loss	5	g.7578457-7578457G>GC
3052	COG	Loss	5	g.7578526-7578526G>GT
3056	COG	Loss	5	g.7578413-7578413G>A¥
3143	SIOP	Loss	5	g.7578394-7578394A>C¥
1142	COG	Loss	8	g.7577120-7577120G>GA¥
1706	SIOP	Loss	8	g.7577120-7577120G>GA¥
2967	SIOP	Loss	8	g.7577129-7577129T>TG
1134†	COG	Loss	8	g.7577118-7577118G>GT¥
1134†	COG	Loss	8	g.7577095-7577095C>CA
1145*	COG	Normal	8	g.7577124C>T¥
3041	COG	Loss	intronic	g.7578298-7578308delCCTCACTGATT
1135	COG	Normal	intronic	g.7578296-7578305het_delTCACTGATTG
9533	SIOP	Unknown	10	g.7574002C>G¥
*Stopgain SNV*				
4718	SIOP	Loss	6	g.7578212-7578212C>CT¥
1136	COG	Loss	10	g.7574003-7574003C>T¥
3145*	SIOP	Loss	6	g.7578263-7578263G>A¥

SNV: Single Nucleotide Variation.

† : Case that had two mutations together with *TP53* loss.

*Cases identified by deep-sequencing.

¥ Mutations found in Li Fraumeni patients (IARC TP53 Database, R17).

The other 18 DAWT samples did not have mutations despite clear morphological evidence of anaplasia in the frozen section. All mutations are described in the IARC TP53 Database (http://www-p53.iarc.fr/) [20]. Loss of *TP53* was identified by MLPA in an additional 3 cases without *TP53* mutation. In total, 20 out of 25 cases (80%) had *TP53* mutation and/or 17p loss.

Several additional alterations were previously reported to be normal variations in the population at frequencies of 0.5–60% (source: dbSNP) and assumed to lack functional consequences (**Table 2**). These variants may have an unknown significance for the patients with AWT, particularly in those three cases with only 17p loss. These were not considered for outcome analysis.

**Table 2:** Variants with unknown function in cases with 17p loss.

Cases	SNP location	SNP	dbSNP	MAF
*Synonymous SNV*				
3043, 3061, 3864	Exon 4	g.7579472-7579472C>G, NM_001126118:c.C98G:p.P33R	rs1042522	0.3979
*Silent mutations*				
3043, 3060, 3061	Exon 11, UTR 3'	g.7572890-7572890A>T	rs1042526	not available
3043, 3061	Exon 2, UTR 5'	g.7579801-7579801C>G	rs1642785	0.3636
3043, 3060, 3061	intronic	g.7579644-7579659del16	not available	

MAF: minor allele frequency (source: dbSNP).

### Mutations in *TP53* effectively risk stratify patients with DAWT

The two cohorts (COG and SIOP) were similar in terms of their clinicopathologic characteristics and overall survival (**Tables S1 and S2**). Whilst this does not preclude that a larger cohort might display different characteristics, outcomes were similar in both trials. Among 32 patients treated in the COG trial, 13 (41%) relapsed and 11 (34%) died, whereas among 8 patients treated in the SIOP trial, 4 relapsed and died (50%). Typically, patients with DAWT relapse within 2 years and only one patient had a follow-up shorter than 5 years (2.39 years); the median follow-up period of patients still alive (n = 25) was 5.92 years.

For the 17 patients (out of 40) that experienced an event, median time from initial diagnosis to tumour recurrence was 0.75 years (range 0.34–1.61 years) and to death was 1.27 years (range 0.5–2.25 years). We defined mutated cases (mut*TP53*) as those 25 with damaging aberrations identified by either sequencing methodology, Sanger (20 cases) or deep-sequencing (2 cases), and/or 17p loss identified by MLPA (23 cases) with an overlap of 21 cases between sequence mutations and locus loss. The other 15 cases were considered wild-type *TP53* (wt*TP53*) cases.

**Figure 2:**
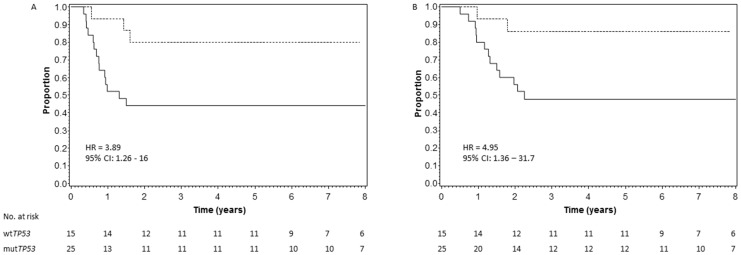
Patients with diffuse anaplastic WT. (A) Event-free survival (p = 0.02) and (B) overall survival curves (p = 0.02) for patients with wt*TP53* (dashed line) versus mut*TP53* (solid line). Numbers at risk are plot every year. HR: hazard ratio, CI: confidence interval.

The 15 patients with wt*TP53* had statistically significantly better EFS than the 25 patients with mut*TP53* (p = 0.02, **Figure 2A**). Five-year EFS estimates were 80% for patient with wt*TP53* and 44% for patients with mut*TP53*. The estimated hazard ratio (HR) for failure comparing mut*TP53* to wt*TP53* was 3.89 (95% profile likelihood confidence interval (CI): 1.26, 16.9).

The results were similar for overall survival. Patients with wt*TP53* had statistically significantly better OS than the patients with mut*TP53* (p = 0.02, **Figure 2B**). Five-year OS estimates were 86% for patients with wt*TP53*, compared to 48% for patients with mut*TP53*. The estimated HR for death comparing mut*TP53* to wt*TP53* was 4.95 (95% profile likelihood CI: 1.36, 31.7). All but one of the 15 deaths was due to tumour progression or relapse. Only a single patient with initial stage I DAWT was known to be alive after relapse (**Table S2**, case 1136). From the 7 stage I/II patients wild type for TP53 only one relapsed, whilst 6 out of 12 stages I/II patients with mutated TP53 relapsed. Unfortunately, the number of patients used in this study doesn't allow us to draw conclusions regarding the association between stage and relapse or TP53 mutation.

### Mutations in *TP53* are associated with genomic instability

To determine if *TP53* mutation was associated with the genomic instability described previously in tumours with anaplasia, copy number alterations (CNAs) were analysed by aCGH in 32 samples. While cases with wt*TP53* (n = 13) had an average of 26.6 (standard deviation (SD) ±18.4) CNAs, cases with mutated *TP53* (n = 19) had a significantly higher average of 44.7 (SD ±26.1) CNAs (**Table S2**). The mean difference was 18.1 (95% CI of difference, 2.0 to 34.2) between groups (p = 0.03, Student's t-test).

### Cell cycle pathways are altered in DAWT with *TP53* mutations

Gene expression profile was compared between 21 DAWT cases with mut*TP53* and 15 DAWT cases with wt*TP53*. This analysis revealed 93 differentially expressed genes (p≤0.01, t-test, **Table S4**), with over-representation of genes that participate in cell cycle: *AURKA*, *RNF8*, *BRCA2*, *RPS27L*, *NOL10*, *RRM2*, *ORC5*, *CCNO*, *HUS1*, *CDKN1A*, *FAM83D*, *PSMC2*, *NCAPH*, *PKP4*, *SUPT5H*, *USP3*, *FGFR10P*, *BUB1*, *TP53*, *MLF1*, *CCNB2*, *PSMB2*, *CDC20* and *SPC25*; and DNA repair processes: *RNF8*, *EGR1*, *RPS27L*, *CCNO*, *HUS1*, *USP3*, *TP53*, *CDKN1A*, *PSMB2*, *PSMC2*, *CCNB2*, *MORF4L2, TNSF11* and *ZMAT3* (**Table S5**). As expected, *TP53* was overexpressed in the wild-type group of samples.

## Discussion

This study aimed to identify how frequently *TP53* mutation underlies DAWT and whether its presence adds additional prognostic information. In this largest set yet described of DAWT, where molecular analysis was confined to DNA extracted from tissue with confirmed anaplastic changes, we found an overall mutation frequency of only 55% (22/40), lower than previously reported [15]–[18], while 17p loss was observed in 58% (23/40) of the cases, with 20 cases showing both *TP53* mutation and 17p loss. Interestingly, from 20 tumours that were *TP53* wild-type by Sanger sequencing, mutations were found in further 2 cases by deep sequencing (162-fold). Hence, only 63% (25/40) of definite DAWT had detectable *TP53* abnormality even when assessed by the most sensitive technology.

A significant association between poor event free and overall survival and *TP53* mutation suggests that it is a potential adverse prognostic factor in addition to anaplastic morphology. The 5-years EFS and OS of patients with DAWT with wt*TP53* were 80% and 86%, respectively, not too dissimilar to the reported EFS and OS of patients with favourable histology tumours treated in the same clinical trials [32]–[34]. However these patients receive more intensive treatment, including doxorubicin, with a predictable risk of long term health problems including cardiac failure and second cancer [35], [36]. It remains to be addressed by future studies and clinical trials if patients with DAWT with and without *TP53* mutations can be classified into different risk categories and have different treatment strategies.

Ten of the mutations we described have been reported in Li-Fraumeni families (**Table 1**), an autosomal dominant syndrome associated with germline *TP53* mutations, characterised by a high incidence of a range of tumours, with WT not being one of the cardinal tumours included in the diagnostic criteria. Some families harbouring *TP53* mutations display a higher WT incidence; five out of six Li-Fraumeni families that developed WT had mutations affecting splicing of *TP53* [37], suggesting an association between risk of these patients to develop WT and the type of *TP53* mutation. Such mutations account for only 4% of all reported germline mutations [38]. Constitutional DNA was not available to exclude the possibility of these mutations being germline.

In the specific biological context of Li-Fraumeni syndrome and medulloblastoma, *TP53* mutations have been associated with chromothripisis, an event characterized by massive genomic rearrangements, causing genomic instability [30], which may be an important evolutionary driver of metastatic ability or chemotherapy treatment resistance [39]. Similar events have been observed in chronic lymphocytic leukaemia [40] and osteosarcomas [41]. We also found that the DAWT with mutated *TP53* had more CNAs than the DAWT with wild-type alleles, indicating that the cells are in a state of genomic instability, likely to be a consequence of the mutated *TP53*. Whether increase in genomic instability is a function of time remains to be addressed.

Hence, unsurprisingly, cell cycle and DNA repair processes were over-represented among the differentially expressed genes. As a cell cycle inhibitory transcription factor, *TP53* regulates genes that enforce its growth inhibitory functions in response to oncogenic or damage-induced stress [42]. *RPS27L* and *CDKN1A*, known TP53 targets, are down-regulated in tumours without wild-type *TP53*, a similar finding to a study that evaluated *TP53* targets across ten cancer types [43]. Together with *TP53*, *CASP8*, *CDKN1A*, *ZMAT3*, *RRM2* and *CCNB2* also belong to the p53 signalling pathway (KEGG), which perturbation is considered a hallmark of cancer [44]. Among these, only *CASP8* has been described in WT. *CASP8*, down-regulated in DAWT with mutated *TP53*, is a pro-apoptotic factor expressed in favourable histology WT, with no correlation to stage of disease or risk for tumour recurrence [45].

The genes representative of the DNA repair pathway found by this study have not been evaluated in WT. Previous studies reported the lack of defects in the DNA mismatch repair system mainly in favourable histology WT based on the pattern of protein expression of *MLH1*, *PMS2*, *MSH2* and *MSH6* [46], [47] and absence of microsatellite instability [47]. Further work will be necessary to clarify which genes and repair pathways are activated in the mutated *TP53* DAWT and at which extension they are important for the other WT subtypes. Analysis of prognostic factors within the histological subgroup of DAWT is challenging, due to its extreme rarity. Less than 10% of Wilms tumour are classified as anaplastic, either focal or diffuse [12]–[14]. Therefore, to obtain a reliable estimate of the frequency of mutated *TP53* in DAWT, we made several efforts: 1) Samples were combined through a collaboration between UK and North American clinical trials. 2) In addition to the rigorous central pathology review process of each group, the frozen samples used for DNA extraction were again reviewed to ensure the presence of anaplasia in the specimen, since an association between the visible anaplastic area and *TP53* mutation is described [15]. 3) Samples that were negative for mutation by conventional sequencing were re-sequenced using a high-coverage methodology (next-generation sequencing). Altogether, these patients and samples were very carefully reanalysed concerning all aspects of their disease and morphology. Such attention to detail regarding sample selection for mutational analysis will, of course, be required where such an assay may be applied in the real life clinical situation in the future.

*TP53* mutational status appears to be an additional indicator of prognosis as it discriminates two classes of anaplastic WT with different outcomes. The value of *TP53* mutation analysis in the diagnostic work-up of patients with DAWT should now be tested in an independent cohort in order to answer the important question of whether DAWT without *TP53* mutations could be a candidate for reduction in therapy. However, it is possible that the relatively good outcomes were the result of the intensive multi-agent therapy used in modern treatment protocols. As we are approaching the limit of tolerability of classical chemotherapy treatment for these patients, it is clear that novel therapeutic strategies are necessary to cure patients with DAWT and mut*TP53*.

## Supporting Information

Table S1 **Clinical information of the patients.**
(PDF)

Table S2 **Detailed clinical and molecular information of the patients with diffuse anaplastic Wilms tumours.**
(PDF)

Table S3 ***TP53*** **alterations identified in all diffuse anaplastic Wilms tumours.** Alterations were classified as damaging (mutation) using ANNOVAR (http://www.openbioinformatics.org/annovar/).
(PDF)

Table S4 **Differentially expressed genes between AWT with and without** ***TP53*** **mutation.**
(PDF)

Table S5 **Biological Process of the 93 differentially expressed genes.** Only classes and parent classes with at least 5 observations in the selected subset and with an 'Observed vs. Expected' ratio of at least 2 genes are shown.
(PDF)
