# Supplementary material for: TP53 Mutational Status Is a Potential Marker for Risk Stratification in Wilms Tumour with Diffuse Anaplasia
Source: PLoS One. 2014 Oct 14;9(10):e109924. doi: 10.1371/journal.pone.0109924 (PMC4196953; doi:10.1371/journal.pone.0109924)
Supplement: Table S1 — Clinical information of the patients. (PDF) [file pone.0109924.s001.pdf]

	Diffuse anaplastic cases (n=40)
**Age at diagnosis (median)**	4.6 years
**Sex (M:F)**	1:3
**Stage I**	11
**II**	8
**III**	16
**IV**	5
**Immediate nephrectomy**	32
**Preoperative chemotherapy**	8
**Relapsed**	17
**Died**	15
***TP53* mutations**	25 (63%)
