# Supplementary material for: TP53 Mutational Status Is a Potential Marker for Risk Stratification in Wilms Tumour with Diffuse Anaplasia
Source: PLoS One. 2014 Oct 14;9(10):e109924. doi: 10.1371/journal.pone.0109924 (PMC4196953; doi:10.1371/journal.pone.0109924)
Supplement: Table S2 — Detailed clinical and molecular information of the patients with diffuse anaplastic Wilms tumours. (PDF) [file pone.0109924.s002.pdf]

| RMH | Clinical Trial | Pre-operative chemotherapy | Chemotherapy regimen | Age at diagnosis (months) | Sex | Stage | Event (months) | Death (months) | *TP53* status | Number of copy number alterations | Used for gene expression analyses |
|---|---|---|---|---|---|---|---|---|---|---|---|
| 1133 | COG | No | EE4A | 47 | Female | I | N/E | N/E | mutTP53 | 40 | yes |
| 1134 | COG | No | EE4A | 99 | Female | I | N/E | N/E | mutTP53 | 54 | yes |
| 1137 | COG | No | I | 27 | Male | II | N/E | N/E | wtTP53 | 42 | yes |
| 1138 | COG | No | I | 56 | Male | II | N/E | N/E | wtTP53 | 56 | yes |
| 1142 | COG | No | I | 44 | Female | II | N/E | N/E | mutTP53 | 7 | yes |
| 1143 | COG | No | I | 72 | Female | III | N/E | N/E | wtTP53 | 6 | yes |
| 1144 | COG | No | I | 17 | Female | III | N/E | N/E | mutTP53 | 36 | yes |
| 1145 | COG | No | I | 49 | Female | III | N/E | N/E | mutTP53 | N/A | yes |
| 1146 | COG | No | I | 71 | Female | III | N/E | N/E | wtTP53 | 38 | yes |
| 1706 | SIOP | Yes | I | 62 | Female | II | N/E | N/E | mutTP53 | N/A | no |
| 2220 | SIOP | Yes | VCCD + RT | 69 | Female | III | N/E | N/E | wtTP53 | N/A | yes |
| 3039 | COG | No | I | 51 | Female | IV | N/E | N/E | wtTP53 | 14 | yes |
| 3041 | COG | No | I | 46 | Female | III | N/E | N/E | mutTP53 | 91 | yes |
| 3045 | COG | No | I | 60 | Female | I | N/E | N/E | wtTP53 | 6 | yes |
| 3047 | COG | No | I | 58 | Female | III | N/E | N/E | mutTP53 | 11 | yes |
| 3050 | COG | No | EE4A | 36 | Female | I | N/E | N/E | wtTP53 | 23 | yes |
| 3055 | COG | No | I | 56 | Female | II | N/E | N/E | wtTP53 | 23 | yes |
| 3057 | COG | No | EE4A | 31 | Female | I | N/E | N/E | wtTP53 | N/A | yes |
| 3058 | COG | No | I | 53 | Female | IV | N/E | N/E | wtTP53 | 11 | yes |
| 3061 | COG | No | I | 32 | Male | III | N/E | N/E | mutTP53 | 101 | yes |
| 3062 | COG | No | I | 46 | Female | IV | N/E | N/E | wtTP53 | 0 | yes |
| 3143 | SIOP | Yes | AVD | 41 | Female | I | N/E | N/E | mutTP53 | 64 | yes |
| 3477 | SIOP | Yes | VCCD + RT | 38 | Female | II | N/E | N/E | mutTP53 | N/A | no |
| 1135 | COG | No | EE4A | 110 | Female | I | 4 | 15 | mutTP53 | 18 | yes |
| 1136 | COG | No | EE4A | 46 | Female | I | 7 | N/E | mutTP53 | 31 | yes |
| 1140 | COG | No | I | 43 | Male | II | 7 | 12 | wtTP53 | 47 | yes |
| 1149 | COG | No | I | 66 | Female | III | 5 | 18 | mutTP53 | 26 | yes |
| 2557 | SIOP | Yes | VCCD + RT | 85 | Female | III | 9 | 11 | mutTP53 | 62 | yes |
| 2967 | SIOP | Yes | VCCD + RT | 54 | Female | III | 5 | 6 | mutTP53 | N/A | yes |

| | | | | | | | | | | | |
|---|---|---|---|---|---|---|---|---|---|---|---|
| 3033 | COG | No | I | 78 | Female | IV | 19 | N/E | wtTP53 | 46 | yes |
| 3036 | COG | No | I | 62 | Female | II | 7 | 11 | mutTP53 | 58 | yes |
| 3043 | COG | No | I | 72 | Female | III | 12 | 25 | mutTP53 | 15 | yes |
| 3044 | COG | No | EE4A | 44 | Male | I | 6 | 14 | mutTP53 | 50 | yes |
| 3048 | COG | No | I | 93 | Female | III | 9 | 16 | mutTP53 | 42 | yes |
| 3052 | COG | No | I | 57 | Male | I | 16 | 24 | mutTP53 | 36 | yes |
| 3053 | COG | No | I | 74 | Male | III | 17 | 22 | wtTP53 | 34 | yes |
| 3056 | COG | No | I | 94 | Male | IV | 11 | 11 | mutTP53 | 76 | yes |
| 3060 | COG | No | EE4A | 44 | Male | I | 8 | 9 | mutTP53 | 32 | yes |
| 3145 | SIOP | Yes | VCCD + RT | 82 | Male | III | 18 | 19 | mutTP53 | N/A | no |
| 4718 | SIOP | Yes | VCCD + RT | 44 | Female | III | 11 | 27 | mutTP53 | N/A | no |

N/E means that patient did not relapse or die by end of study or loss to follow up. N/A means that CGH-array was not available. The calculation of copy number alterations is defined in the material and methods section.
