# Supplementary material for: TP53 Mutational Status Is a Potential Marker for Risk Stratification in Wilms Tumour with Diffuse Anaplasia
Source: PLoS One. 2014 Oct 14;9(10):e109924. doi: 10.1371/journal.pone.0109924 (PMC4196953; doi:10.1371/journal.pone.0109924)
Supplement: Table S3 — TP53 alterations identified in all diffuse anaplastic Wilms tumours. Alterations were classified as damaging (mutation) using ANNOVAR (http://www.openbioinformatics.org/annovar/). (PDF) [file pone.0109924.s003.pdf]

| DNA Alteration | Patient ID | Localization | Other info | Mapping of alterations (Hg19) | Mutation |
|---|---|---|---|---|---|
| **Frameshift deletion** | | | | | |
| **ALT1** | 1149 | Ex 10 | 10-bp deletion | g.7573999-7574008het_delTGTTCCGAGA | yes |
| **ALT2** | 3036 | Ex 10 | 7-bp deletion | g.7574000-7574006delTTCCGAG | yes |
| **ALT3** | 3048 | Ex 10 | 1-bp deletion | g.7574013-7574013het_delC | yes |
| **Frameshift insertion** | | | | | |
| **ALT4** | 3477 | Ex 11 | 1-bp insertion | g.7572933-7572933dupA | yes |
| **Non-frameshift deletion** | | | | | |
| **ALT5** | 1144 | Ex 6 | 3-bp deletion | g.7578275-7578277delCTC | yes |
| **ALT6** | 1133 | Ex 10 | 6-bp insertion | g.7574026-7574026dupGGCGTG | yes |
| **ALT7** | 2557 | Ex 5 | 27-bp insertion | g.7578447-7578447dupCATCTACAAGCAGTCACAGCACATGAC | yes |
| **Non-synonymous SNV** | | | | | |
| **ALT8** | 1133, 1134, 1135, 1136, 1137, 1138, 1140, 1142, 1143, 1145, 1146, 1706, 2220, 2557, 2967, 3033, 3039, 3041, 3043, 3044, 3045, 3047, 3048, 3050, | Ex 4 | Single substitution | g.7579472-7579472C>G | no |

| | | | | | |
|---|---|---|---|---|---|
| | 3053, 3057, 3058, 3061, 3062, 3143, 4718 | | | | |
| **ALT9** | 3143 | Ex 5 | Single substitution | g.7578394-7578394A>C | yes |
| **ALT10** | 3044 | Ex 5 | Single substitution | g.7578406-7578406G>GA | yes |
| **ALT11** | 3056 | Ex 5 | Single substitution | g.7578413-7578413G>A | yes |
| **ALT12** | 3047 | Ex 5 | Single substitution | g.7578457-7578457G>GC | yes |
| **ALT13** | 3052 | Ex 5 | Single substitution | g.7578526-7578526G>GT | yes |
| **ALT14** | 1134 | Ex 8 | Single substitution | g.7577095-7577095C>CA | yes |
| **ALT15** | 1134 | Ex 8 | Single substitution | g.7577118-7577118G>GT | yes |
| **ALT16** | 1142, 1706 | Ex 8 | Single substitution | g.7577120-7577120G>GA | yes |
| **ALT17** | 2967 | Ex 8 | Single substitution | g.7577129-7577129T>T/G | yes |
| **Splicing mutation** | | | | | |
| **ALT18** | 1135 | intronic | 10-bp deletion | g.7578296-7578305het_delTCACTGATTG | yes |
| **ALT19** | 3041 | intronic | 11-bp deletion | g.7578298-7578308delCCTCACTGATT | yes |
| **Stop-gain SNV** | | | | | |
| **ALT20** | 1136 | Ex 10 | Single substitution | g.7574003-7574003C>T | yes |
| **ALT21** | 4718 | Ex 6 | Single substitution | g.7578212-7578212C>CT | yes |
| **Synonymous SNV** | | | | | |
| **ALT22** | 1133, 1134, 1136, 1146, 1149, 2557, 3044, 3055, 3058, 3062, 3143, 4718 | Ex 3 | Single substitution | g.7579579-7579579G>GA | no |

| | | | | | |
|---|---|---|---|---|---|
| **Silent mutations** | | | | | |
| **ALT23** | 1133, 1134, 1135, 1136, 1137, 1138, 1140, 1142, 1143, 1144, 1145, 1146, 1149, 1706, 2220, 2557, 2967, 3033, 3036, 3043, 3044, 3045, 3047, 3048, 3050, 3052, 3053, 3055, 3056, 3057, 3058, 3060, 3061, 3062, 3143 | Ex 11, UTR3 | Single substitution | g.7572890-7572890A>T | no |
| **ALT24** | 1133, 1134, 1136, 1137, 1138, 1140, 1142, 1143, 1145, 1706, 2220, 2557, 2967, 3033, 3039, 3041, 3043, 3044, 3045, 3047, 3048, 3052, 3053, 3057, 3058, 3061, 3062, 3143, 4718 | Ex 2, UTR5 | Single substitution | g.7579801-7579801C>G | no |
| **ALT25** | 1135, 3050 | intronic | Single substitution | g.7576841-7576841T>TC | no |
| **ALT26** | 1135 | intronic | Single substitution | g.7577188-7577188T>TC | no |
| **ALT27** | 1146 | intronic | Single substitution | g.7579596-7579596C>CT | no |
| **ALT28** | 1146 | intronic | Single substitution | g.7579596-7579596C>CT | no |
| **ALT29** | 1140, 1146, 3477 | intronic | Single substitution | g.7579619-7579619C>CA | no |
| **ALT30** | 1133, 1134, 1136, 1137, 1138, 1140, 1142, 1143, 1145, 1146, 1149, 1706, 2220, 2557, 3033, 3036, 3039, 3041, 3043, 3044, 3045, 3047, 3048, 3050, 3052, 3053, 3055, 3056, 3057, 3058, 3060, 3061, 3062, 3143, 3477, 4718 | intronic | 16-bp deletion | g.7579644-7579659del16 | no |
