# Supplementary material for: TP53 Mutational Status Is a Potential Marker for Risk Stratification in Wilms Tumour with Diffuse Anaplasia
Source: PLoS One. 2014 Oct 14;9(10):e109924. doi: 10.1371/journal.pone.0109924 (PMC4196953; doi:10.1371/journal.pone.0109924)
Supplement: Table S4 — Differentially expressed genes between AWT with and without TP53 mutation. (PDF) [file pone.0109924.s004.pdf]

Symbol	Parametric p-value	Fold-change mut*TP53* x wt*TP53*	Geometric mean of ratios in mut*TP53*	Geometric mean of ratios in *wtTP53*
NRN1	0.00	2.35	0.40	0.17
Hs.47208	0.00	2.12	2.76	1.30
AURKA	0.00	2.00	0.39	0.20
RRM2	0.00	1.94	0.46	0.24
GPR158	0.00	1.93	1.58	0.81
C20orf129	0.00	1.90	0.62	0.33
clone 3347440	0.00	1.87	0.11	0.06
MGC44505	0.00	1.85	0.83	0.45
NR6A1	0.01	1.83	0.21	0.11
CCNB2	0.01	1.81	0.64	0.35
Hs.184938	0.00	1.78	0.84	0.47
BUB1	0.01	1.71	0.66	0.39
PHGDH	0.01	1.71	0.51	0.30
SSR1	0.01	1.66	0.35	0.21
PEX5R	0.01	1.65	1.58	0.96
CEL	0.01	1.63	0.27	0.16
CDC20	0.01	1.62	0.50	0.31
GDAP2	0.00	1.62	0.92	0.57
DESR1	0.00	1.60	0.76	0.47
MLF1	0.01	1.57	1.90	1.21
BRRN1	0.00	1.56	0.60	0.39
ALDH1A1	0.00	1.55	1.24	0.80
GRIN1	0.01	1.55	1.12	0.72
PSMB2	0.01	1.54	0.84	0.54
KIF4A	0.00	1.53	0.77	0.51
PSMC2	0.00	1.52	0.71	0.47
C14orf92	0.00	1.51	0.97	0.64
FLJ23469	0.00	1.51	0.89	0.59
KIAA0090	0.00	1.50	0.66	0.44
FGFR1OP	0.00	1.48	0.87	0.59
MORF4L2	0.01	1.47	0.66	0.45
SPC25	0.01	1.47	0.45	0.30
HS2ST1	0.00	1.46	1.47	1.01
LOC126731	0.00	1.46	0.84	0.58
SATB2	0.01	1.46	0.77	0.53
LOC100129675	0.00	1.45	0.77	0.53
TAF6L	0.01	1.45	0.30	0.21
SPRR2C	0.01	1.44	1.20	0.83
ITCH	0.00	1.43	0.75	0.52
PBX2	0.01	1.39	1.43	1.03
UNG2	0.00	1.39	1.56	1.12
MRPS10	0.00	1.38	0.65	0.47
TNPO3	0.00	1.37	0.70	0.51
C7orf10	0.01	1.36	1.24	0.91
PFDN4	0.00	1.36	0.97	0.71
SUPT5H	0.00	1.36	1.31	0.96
FLJ10786	0.00	1.35	0.91	0.68

ORC5L	0.00	1.35	0.62	0.46
TNFSF11	0.01	1.35	1.23	0.91
KCMF1	0.00	1.34	0.76	0.57
SCAMP3	0.00	1.34	0.81	0.60
BXDC1	0.00	1.33	0.63	0.47
HUS1	0.00	1.33	0.92	0.69
MADHIP	0.01	1.33	1.40	1.05
PKP4	0.00	1.32	0.76	0.58
RNF8	0.00	1.32	1.19	0.90
TAF12	0.01	1.32	0.69	0.53
CGI-94	0.01	1.31	0.58	0.44
eIF3k	0.01	1.27	0.95	0.75
FLJ21174	0.01	1.27	1.17	0.92
FLJ14075	0.00	1.25	0.82	0.65
USP3	0.00	1.24	0.91	0.74
FLJ11193	0.01	1.23	0.92	0.75
C20orf126	0.01	1.22	1.24	1.02
DKFZP434L1717	0.01	1.21	0.84	0.70
DHODH	0.01	-1.09	0.90	0.98
GCGR	0.01	-1.25	1.03	1.30
SMYD4	0.00	-1.27	0.90	1.13
CASP8	0.01	-1.28	0.91	1.16
MGC34732	0.00	-1.28	0.88	1.13
LOC283130	0.01	-1.30	0.71	0.92
GFRA3	0.00	-1.32	0.75	0.98
C20orf81	0.00	-1.33	1.08	1.44
CGI-150	0.00	-1.33	0.94	1.26
C1RL	0.00	-1.39	1.01	1.40
MPDU1	0.00	-1.39	0.70	0.97
ZNF18	0.01	-1.39	0.82	1.13
PAEP	0.00	-1.41	0.55	0.78
TOM1L2	0.00	-1.41	0.76	1.07
Hs.262480	0.01	-1.43	0.97	1.39
NLGN2	0.00	-1.43	0.76	1.10
PHLDB1	0.00	-1.43	1.33	1.91
FLJ13955	0.00	-1.49	0.91	1.37
SFRS5	0.01	-1.49	0.63	0.95
RPS27L	0.00	-1.54	0.71	1.09
GABARAP	0.00	-1.56	1.26	1.97
KIAA0590	0.00	-1.56	0.93	1.45
TP53	0.01	-1.64	0.84	1.36
EGR1	0.01	-1.69	0.51	0.87
Hs.437875	0.00	-1.69	1.56	2.64
LIP8	0.00	-1.69	0.54	0.91
ZMAT3	0.00	-1.82	1.05	1.89
CDKN1A	0.00	-2.12	0.49	1.04
