# Supplementary material for: TP53 Mutational Status Is a Potential Marker for Risk Stratification in Wilms Tumour with Diffuse Anaplasia
Source: PLoS One. 2014 Oct 14;9(10):e109924. doi: 10.1371/journal.pone.0109924 (PMC4196953; doi:10.1371/journal.pone.0109924)
Supplement: Table S5 — Biological Process of the 93 differentially expressed genes. Only classes and parent classes with at least 5 observations in the selected subset and with an 'Observed vs. Expected' ratio of at least 2 genes are shown. (PDF) [file pone.0109924.s005.pdf]

GO ID	GO Term	Observed in selected subset	Expected in selected subset	Observed/ Expected
GO:0006284	base-excision repair	5	0.39	12.86
GO:0007569	cell aging	5	0.43	11.66
GO:0044783	G1 DNA damage checkpoint	7	0.63	11.11
GO:0031571	mitotic G1 DNA damage checkpoint	7	0.63	11.11
GO:2000134	negative regulation of G1/S transition of mitotic cell cycle	7	0.66	10.66
GO:0044773	mitotic DNA damage checkpoint	7	0.67	10.44
GO:0044774	mitotic DNA integrity checkpoint	7	0.68	10.24
GO:2000045	regulation of G1/S transition of mitotic cell cycle	7	0.72	9.67
GO:0030330	DNA damage response, signal transduction by p53 class mediator	5	0.86	9.32
GO:0072431	signal transduction involved in mitotic G1 DNA damage checkpoint	5	0.55	9.1
GO:0072422	signal transduction involved in DNA damage checkpoint	5	0.55	9.1
GO:0072413	signal transduction involved in mitotic cell cycle checkpoint	5	0.55	9.1
GO:0072401	signal transduction involved in DNA integrity checkpoint	5	0.55	9.1
GO:0072395	signal transduction involved in cell cycle checkpoint	5	0.55	9.1
GO:0006977	DNA damage response, signal transduction by p53 class mediator resulting in cell cycle arrest	5	0.55	9.1
GO:0071158	positive regulation of cell cycle arrest	5	0.56	8.88
GO:0071156	regulation of cell cycle arrest	5	0.59	8.48
GO:0042770	signal transduction in response to DNA damage	8	0.95	8.4
GO:0000077	DNA damage checkpoint	8	1.03	7.75
GO:0031570	DNA integrity checkpoint	8	1.1	7.28
GO:0072527	pyrimidine-containing compound metabolic process	6	0.94	6.39
GO:0000082	G1/S transition of mitotic cell cycle	9	1.43	6.27
GO:0010212	response to ionizing radiation	5	0.8	6.22
GO:0031145	anaphase-promoting complex-dependent proteasomal ubiquitin-dependent protein catabolic process	8	1.3	6.15
GO:0007568	aging	6	1.09	5.53
GO:0090068	positive regulation of cell cycle process	5	1.01	4.97
GO:0051439	regulation of ubiquitin-protein ligase activity involved in mitotic cell cycle	9	1.82	4.94
GO:0006354	DNA-dependent transcription, elongation	5	1.06	4.72
GO:0009314	response to radiation	9	1.92	4.69
GO:0022402	cell cycle process	68	15.35	4.43
GO:0000278	mitotic cell cycle	51	11.66	4.37
GO:0007049	cell cycle	93	22.55	4.12
GO:0043161	proteasomal ubiquitin-dependent protein catabolic process	8	2.21	3.62
GO:0010498	proteasomal protein catabolic process	8	2.25	3.55

GO:0048285	organelle fission	9	2.65	3.39
GO:0007067	mitosis	8	2.52	3.17
GO:0000280	nuclear division	8	2.52	3.17
GO:0001558	regulation of cell growth	5	1.73	2.89
GO:0000226	microtubule cytoskeleton organization	6	2.16	2.78
GO:0031400	negative regulation of protein modification process	6	2.35	2.56
GO:0016042	lipid catabolic process	5	1.98	2.52
GO:0007017	microtubule-based process	8	3.3	2.43
GO:0043086	negative regulation of catalytic activity	7	3.02	2.32
GO:0016570	histone modification	6	2.65	2.26
GO:0016569	covalent chromatin modification	6	2.68	2.24
GO:0045944	positive regulation of transcription from RNA polymerase II promoter	5	2.24	2.23

Biological processes directly associated with DNA repair pathways are highlighted in red.
